# Supplementary material for: PD-L1 Expression and Tumor-Infiltrating Lymphocytes in Thymic Epithelial Neoplasms
Source: J Clin Med. 2019 Nov 1;8(11):1833. doi: 10.3390/jcm8111833 (PMC6912585; doi:10.3390/jcm8111833)
Supplement: Supplementary file 1 [file jcm-08-01833-s001.pdf]

Journal of
Clinical Medicine

MDPI

Table S1. Detailed clinicopathological features and immunophenotypic data of individual patients.

Case No.	Case No. in Table 1	Sex	Age (years)	Smoking habit	MG	Surgical procedure	WHO histology	Tumor size (cm)	Masaoka stage	Fixing method	PD-L1 staining (%)	CD8/CD3 (%)
1	26	M	78	smoker		extended thymectomy	B3	10.0	II	20% non-buffered	0	90
2	27	F	64	non-smoker		extended thymectomy	B3	10.7	III	20% non-buffered	70	70
3	32	F	47	non-smoker		extended thymectomy	C	7.0	III	20% non-buffered	40	40
4	1	M	60	smoker		extended thymectomy	A	6.0	I	20% non-buffered	0	90
5	33	M	57	smoker		biopsy	C	-	III	20% non-buffered	0	90
6	13	F	30	non-smoker		extended thymectomy	B1	7.0	II	20% non-buffered	0	70
7	28	F	75	non-smoker		extended thymectomy	B3	2.0	III	20% non-buffered	80	40
8	34	M	53	smoker		biopsy	C	-	III	20% non-buffered	90	90
9	14	M	55	smoker		extended thymectomy	B1	10.0	I	20% non-buffered	70	90
10	15	F	49	non-smoker		tumor resection	B1	6.0	I	20% non-buffered	0	90
11	35	F	85	non-smoker		biopsy	C	-	III	20% non-buffered	0	90
12	7	F	59	non-smoker		tumor resection	AB	4.5	II	20% non-buffered	0	90
13	16	F	57	non-smoker		extended thymectomy	B1	7.0	I	20% non-buffered	0	50
14	8	M	63	smoker		extended thymectomy	AB	12.5	I	20% non-buffered	0	90
15	36	M	63	smoker		extended thymectomy	C	8.5	IVa	20% non-buffered	0	90
16	17	F	85	non-smoker		biopsy	B1	-	IVa	20% non-buffered	70	90
17	9	M	60	smoker		extended thymectomy	AB	6.0	I	20% non-buffered	0	90
18	29	M	23	non-smoker		extended thymectomy	B3	9.0	I	20% non-buffered	60	90
19	30	M	43	non-smoker		extended thymectomy	B3	6.0	I	20% non-buffered	90	5
20	37	M	76	smoker		biopsy	C	-	IVa	20% non-buffered	30	90
21	18	F	42	non-smoker		extended thymectomy	B1	3.0	II	10% buffered	0	90
22	10	M	76	smoker		extended thymectomy	AB	5.5	II	10% buffered	3	90
23	38	F	82	non-smoker		extended thymectomy	C	6.0	II	10% buffered	70	70
24	22	M	76	smoker	+	tumor resection	B2	2.0	I	10% buffered	70	90
25	2	F	68	non-smoker		extended thymectomy	A	4.5	II	10% buffered	80	90
26	39	F	66	smoker		extended thymectomy	C	5.0	IVa	10% buffered	5	90
27	19	F	76	smoker		extended thymectomy	B1	9.0	II	10% buffered	1	90
28	3	M	71	smoker		extended thymectomy	A	3.0	I	10% buffered	0	90
29	4	M	65	smoker		extended thymectomy	A	2.5	I	10% buffered	0	90
30	23	M	65	non-smoker		extended thymectomy	B2	4.0	II	10% buffered	70	90
31	31	M	81	smoker		extended thymectomy	B3	4.0	III	10% buffered	90	90

32	5	F	80	non-smoker	extended thymectomy	A	4.5	III	10% buffered	0	90
33	20	F	48	smoker	extended thymectomy	B1	2.5	II	10% buffered	0	95
34	11	M	62	smoker	tumor resection	AB	4.0	I	10% buffered	0	90
35	21	M	73	non-smoker	extended thymectomy	B1	10.0	II	10% buffered	0	90
36	6	M	65	smoker	extended thymectomy	A	3.0	I	10% buffered	30	90
37	12	F	45	non-smoker	extended thymectomy	AB	5.0	I	10% buffered	10	90
38	24	F	53	smoker	extended thymectomy	B2	2.1	I	10% buffered	50	90
39	25	M	67	smoker	biopsy	B2	10.0	IVa	10% buffered	70	90

M, male; F, female; MG, myasthenia gravis.

© 2019 by the authors. Licensee MDPI, Basel, Switzerland. This article is an open access article distributed under the terms and conditions of the Creative Commons Attribution (CC BY) license (http://creativecommons.org/licenses/by/4.0/).
